# Silver Nanoparticles Using Eucalyptus or Willow Extracts (AgNPs) as Contact Lens Hydrogel Components to Reduce the Risk of Microbial Infection

**DOI:** 10.3390/molecules26165022

**Published:** 2021-08-19

**Authors:** Andreas K. Rossos, Christina N. Banti, Panagiotis K. Raptis, Christina Papachristodoulou, Ioannis Sainis, Panagiotis Zoumpoulakis, Thomas Mavromoustakos, Sotiris K. Hadjikakou

**Affiliations:** 1 Section of Inorganic and Analytical Chemistry, Department of Chemistry, University of Ioannina, 45110 Ioannina, Greece; arossos@uoi.gr (A.K.R.); panagiwtisraptis93@yahoo.gr (P.K.R.); 2 Department of Physics, University of Ioannina, 45110 Ioannina, Greece; xpapaxri@uoi.gr; 3 Cancer Biobank Center, University of Ioannina, 45110 Ioannina, Greece; isainis@uoi.gr; 4 Laboratory of Chemistry, Analysis and Design of Food Processes, Department of Food Science and Technology, University of West Attica, 12243 Attica, Greece; pzoump@eie.gr; 5 Organic Chemistry Laboratory, Department of Chemistry, University of Athens Greece, 15571 Athens, Greece; tmavrom@chem.uoa.gr; 6 University Research Center of Ioannina (URCI), Institute of Materials Science and Computing, 45110 Ioannina, Greece

**Keywords:** silver nanoparticles, eucalyptus leaves extract, willow bark extract, antimicrobial materials, hydrogels, contact lens

## Abstract

Eucalyptus leaves (ELE) and willow bark (WBE) extracts were utilized towards the formation of silver nanoparticles (AgNPs(ELE), AgNPs(WBE)). AgNPs(ELE) and AgNPs(WBE) were dispersed in polymer hydrogels to create pHEMA@AgNPs(ELE)_2 and pHEMA@AgNPs(WBE)_2 using hydroxyethyl-methacrylate (HEMA). The materials were characterized in a solid state by X-ray fluorescence (XRF) spectroscopy, X-ray powder diffraction analysis (XRPD), thermogravimetric differential thermal analysis (TG-DTA), differential scanning calorimetry (DTG/DSC) and attenuated total reflection spectroscopy (ATR-FTIR) and ultraviolet visible (UV-vis) spectroscopy in solution. The antimicrobial potential of the materials was investigated against the Gram-negative bacterial strain *Pseudomonas aeruginosa* (*P. aeruginosa*) and the Gram-positive bacterial strain of the genus *Staphylococcus epidermidis* (*S. epidermidis*) and *Staphylococcus aureus* (*S. aureus*), which are involved in microbial keratitis. The percentage of bacterial viability of *P. aeruginosa* and *S. epidermidis* upon their incubation over the pHEMA@AgNPs(ELE)_2 discs is interestingly low (28.3 and 6.8% respectively), while the inhibition zones (IZ) formed are 12.3 ± 1.7 and 13.2 ± 1.2 mm, respectively. No in vitro toxicity of this material towards human corneal epithelial cells (HCEC) was detected. Despite its low performance against *S. aureus*, pHEMA@AgNPs(ELE)_2 could be an efficient candidate towards the development of contact lenses that reduces microbial infection risk.

## 1. Introduction

Contact lenses have been widely used in recent decades [1,2,3,4]. However, they are associated with bacterial infections such as microbial keratitis (MK) [1,2,3,4]. Bacteria such as *P. aeruginosa, S. epidermidis* and *S. aureus* often colonize materials such as contact lenses [2,3]. This means that there is a requirement for novel materials that can retain the benefits of typical contact lenses without the peril of severe bacterial infections [5,6]. Contact lenses are typically made from hydrogels based on pHEMA; this gives contact lenses the desirable water and oxygen permeability [7,8]. The dispersion of an antimicrobial agent gives antibacterial properties to the polymeric material [5,6,9].

**Scheme 1:**
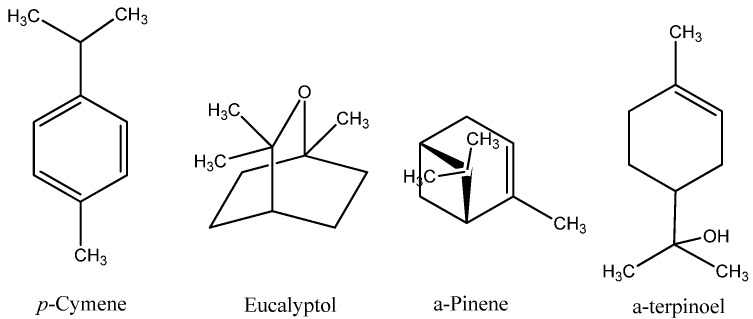
Molecular formulae of the main components of Eucalyptus [11,12].

Plant extracts are used as reducing of capping agents for the chemical synthetic procedure of nanomaterials [10]. The type of plant extract used is known to influence the characteristics of the nanoparticles produced, since different plant extracts contain different concentrations of organic reducing agents [10]. Eucalyptus leaves contain a wide range of active components such as *p*-cymene (42.1%), eucalyptol (1,8-cineole) (14.1%), α-pinene (12.7%) and α-terpineol (10.7%) (Scheme 1) [11,12]. The extract from the leaves or essential oil from the leaves of *Eucalyptus spp*. has been reported to possess antifungal, antibacterial and antioxidant properties [13].

**Scheme 2:**
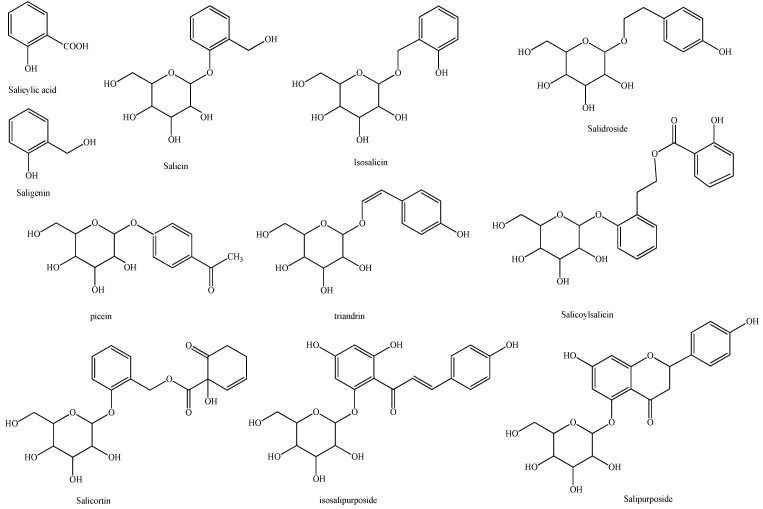
Molecular formulae of the main components of willow bark extract [15].

Willow species extract, deriving either from bark or leaves, has already been used against bacteria (*A. alternaria, A. niger, M. hiemalis, P. notatum* and *S. commune*) with remarkable effect [14]. Analysis of aqueous extracts of willow bark has shown the presence of at least 11 related salicylate compounds including salicin, saligenin, salicylic acid, isosalicin, salidroside, picein, triandrin, salicoylsalicin, salicortin, isosalipurpuroside and salipurpuroside (Scheme 2) [15].

Silver compounds are known antimicrobial agents [9,16]. Recently, the metal–organic framework (MOF) of formula {[Ag_6_(*μ*_3_-HMNA)_4_(*μ*_3_-MNA)_2_]^2−^·[(Et_3_NH)^+^]_2_·2(DMSO)·(H_2_O)} (AGMNA) (H_2_MNA = 2 mercapto-nicotinic acid) has been successfully incorporated in polymer hydrogel pHEMA, providing antimicrobial contact lenses [5]. Silver nanoparticles, on the other hand, constitute an alternative way to overcome antimicrobial resistance [17]. Recently, the synthesis of silver nanoparticles, using eucalyptus leaf extract, and their antimicrobial activity against two Gram-negative bacteria (*P. aeruginosa* and *E. coli*) and two Gram-positive bacteria (*S. aureus* and *B. subtilis*) have been reported [12,18].

Two strategies have been developed, with the aim of forming new antimicrobial materials for contact lenses that are able to reduce microbial infection risk. One involves the usage of silver nanoparticles using extracts from natural products as a combined reducing and capping agent [6] while the second is the use of small molecules, which also act as antimicrobial agents [5,6,9,19]. The materials pHEMA@ORLE_2 and pHEMA@AgNPs(ORLE)_2, which contain silver nanoparticles AgNPs(ORLE) from oregano leaves’ extract (ORLE), were reported on recently [6]. The antimicrobial ability of these materials against the bacteria that are involved in the microbial contamination of contact lenses demonstrates the value of further research into this strategy [6]. In continuing our work in this research area [6], silver nanoparticles (AgNPs(ELE), AgNPs(WBE)) using eucalyptus leaves (ELE) and willow bark (WBE) extracts were synthesized, and were incorporated in pHEMA hydrogels, forming the materials pHEMA@AgNPs(ELE)_2 and pHEMA@AgNPs(WBE)_2. The materials were characterized by XRF, XRPD, TG-DTA, DTG/DSC, ATR-FTIR and UV-vis spectroscopy. The antimicrobial potential of the materials was evaluated against the Gram-negative bacterial strain *P. aeruginosa* and the Gram-positive ones, *S. epidermidis* and *S. aureus*, which are involved in the microbial contamination of contact lenses, leading to MK.

## 2. Results

### 2.1. General Aspects

pHEMA cross-linked with ethylene glycol dimethacrylate (EGDMA) is the basis of many types of soft contact lenses made for daily use [5]. In order to evaluate the antimicrobial efficiency of the contact lens (pHEMA@AgNPs(ELE)_2 and pHEMA@AgNPs(WBE)_2), the AgNPs(ELE) or AgNPs(WBE) nanoparticles were dispersed in pHEMA in a concentration of 2 mg/mL during the polymerization procedure [6]. Discs of the materials, 10 mm in diameter, were cut, cleaned from monomers and stored either in sterilized NaCl 0.9% *w*/*w* solution or they dried at 50 °C. 

### 2.2. X-ray Fluorescence Spectroscopy

**Figure 1:**
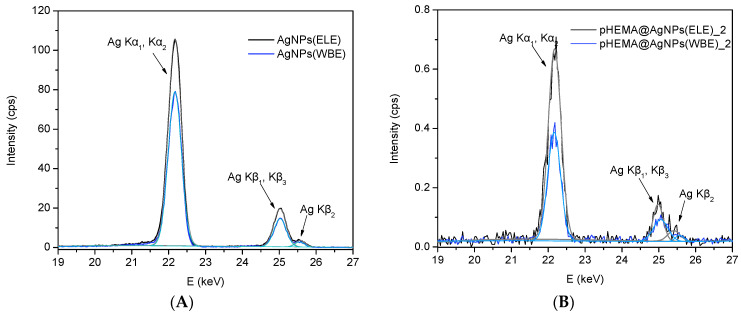
XRF spectra of AgNPs(ELE), AgNPs(WBE) (**A**), pHEMA@AgNPs(ELE)_2 and pHEMA@AgNPs(WBE)_2 (**B**). The Ag K_a_ was used to determine Ag content within the samples.

Dry pHEMA@AgNPs(ELE)_2 and pHEMA@AgNPs(WBE)_2 discs were ground to powder. The XRF spectrum of AgNPs(ELE), AgNPs(WBE), pHEMA@AgNPs(ELE)_2 and pHEMA@AgNPs(WBE)_2 powders confirmed the presence of Ag in the materials (Figure 1). The content of silver in AgNPs(ELE), AgNPs(WBE), pHEMA@AgNPs(ELE)_2 and pHEMA@AgNPs(WBE)_2 was determined at 46 ± 7, 32 ± 5, 0.30 ± 0.04 and 0.20 ± 0.03% *w*/*w*, respectively. The latter suggests the successful encapsulation of AgNPs within pHEMA.

### 2.3. X-ray Powder Diffraction Analysis (XRPD)

**Figure 2:**
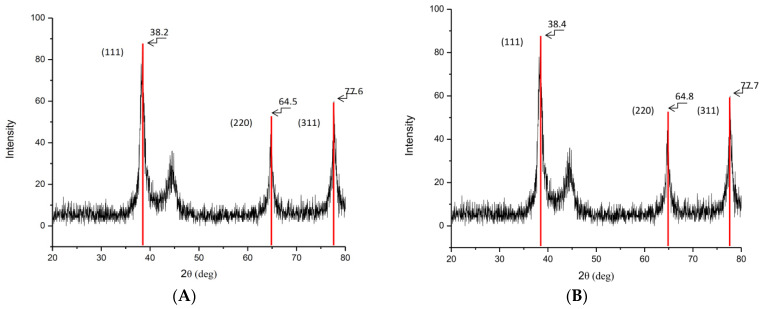
Powder X-ray diagram of AgNPs(ELE) (**A**) and AgNPs(WBE) (**B**) with the characteristic Bragg peaks of Ag nanoparticles.

The crystallite size of AgNPs(ELE) and AgNPs(WBE) was determined with XRPD (Figure 2). The Voigt shape function was used during the peak fitting. The XRPD pattern clearly showed the main peaks at (2θ) 38.2, 44.4, 64.5 and 77.6° (AgNPs(ELE)) corresponding to the (111), (200), (220) and (311) planes, as well as at (2θ) 38.4, 64.8 and 77.7° corresponding to the (111), (200), (220) and (311) planes (AgNPs(WBE)), respectively. The silver nanoparticles were face-centered, cubic and crystalline in nature [20] (correlated to JCPDS card: number 04-0783) [21].

Peak width was defined as the full width at half maximum (FWHM), knowing that the peak width varies inversely with crystallite size. The average crystalline size of the silver nanoparticles was estimated using the Debye–Scherrer’s Equation (1) [22]

B(2θ) = 0.9 × λ/(L × cos(θ))(1)

where B = peak width, which is inversely proportional to crystallite size (L).

The profile of the three Bragg peaks at 38.2, 64.5 and 77.6° (AgNPs(ELE)) and at (2θ) 38.4, 64.8 and 77.7° (AgNPs(WBE)) were calculated, and the following corresponding crystallite sizes were obtained: AgNPs(ELE), 21.3, 15.0 and 11.1 nm (or 213, 150, 111 Å) and for AgNPs(WBE)), 7.9, 11.5 and 7.8 nm (or 79, 115, 78 Å). Therefore, the average crystallite size values are 15.8 nm (AgNPs(ELE)) and 9.1 nm (AgNPs(WBE)).

### 2.4. Thermo Gravimetric Analysis of AgNPs(ELE), AgNPs(WBE), pHEMA, pHEMA@AgNPs(ELE)_2 and pHEMA@AgNPs(WBE)_2

#### 2.4.1. Differential Scanning Calorimetry (DSC)

**Figure 3:**
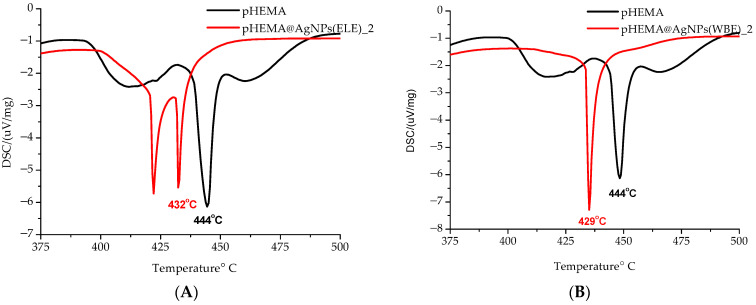
Differential scanning calorimetry (DSC) thermo-diagrams of pHEMA, pHEMA@AgNPs(ELE)_2 (**A**) and pHEMA@AgNPs(WBE)_2 (**B**).

In order to ascertain whether AgNPs(ELE) and AgNPs(WBE) nanoparticles interact with pHEMA in a solid state, towards a mixture or a composite material, DSC studies on the powder of dry pHEMA and pHEMA@AgNPs(ELE)_2 or pHEMA@AgNPs(WBE)_2 were performed (Figure 3). The endothermic transitions in the DSC diagram of the free pHEMA at 444 °C is observed at 432 °C pHEMA@AgNPs(ELE)_2 and at 429 °C in pHEMA@AgNPs(WBE)_2, respectively, implying an interaction between the material’s components with pHEMA and therefore the formation of composite materials.

#### 2.4.2. Thermal Decomposition

TG/DTA analysis was performed under air on the powder of dry AgNPs(ELE), AgNPs(WBE), pHEMA@AgNPs(ELE)_2 and pHEMA@AgNPs(WBE)_2 with an increasing temperature rate of 10 °C min^−1^ from ambient up to 500 °C (Figure S1). The silver nanoparticles AgNPs(ELE) decomposes with three endothermic steps at 55–90, 90–370 and 427–450 °C, while the and AgNPs(WBE) with six ones at 50–85, 85–248, 248–404, 404–452,452–478 and 478–510 °C and (AgNPs(WBE)) (Figure S1). The composite materials pHEMA@AgNPs(ELE)_2 and pHEMA@AgNPs(WBE)_2 decompose with three endothermic steps at 55–160, 243–380 and 427–450 °C (pHEMA@AgNPs(ELE)_2) and at 55–135, 230–395 and 429–484 °C (pHEMA@AgNPs(WBE)_2) (Figure S1).

### 2.5. Attenuated Total Reflection Spectroscopy (ATR-FTIR)

**Figure 4:**
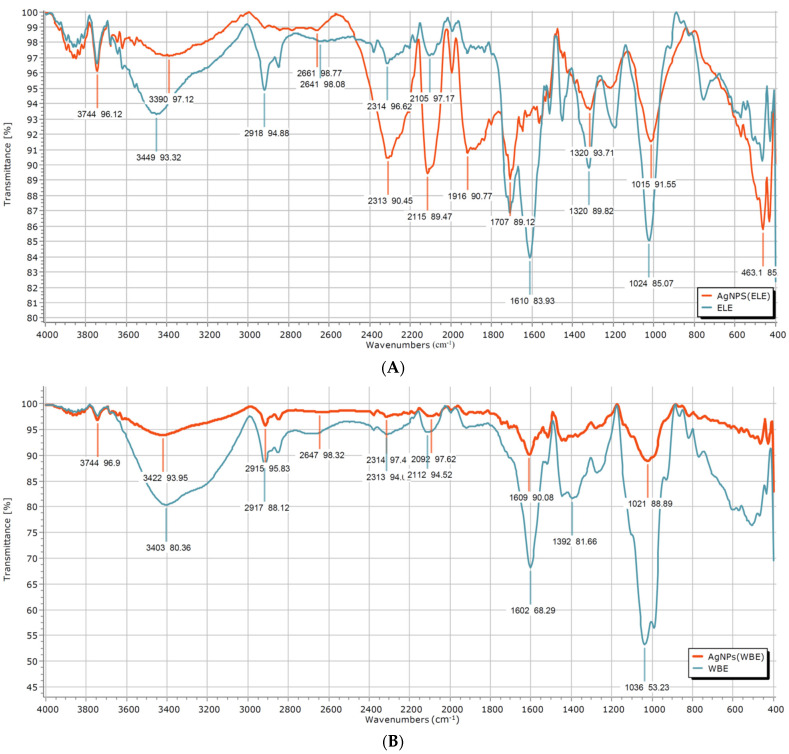
ATR-FTIR specra of ELE, AgNPs(ELE) (**A**) and WBE, AgNPs(WBE) (**B**).

ATR-FTIR spectra of AgNPs(ELE) and AgNPs(WBE) exhibited the characteristic stretching vibration of bands of the alcohols and phenolic groups of plant extracts, which also appeared on the spectra of free extracts (Figure 4). The C-H stretching vibration band at 2918 cm^−1^ (ELE or WBE), the C-OH band at 1024 or 1036 cm^−1^ (ELE or WBE) and the aromatic C=C vibration band at 1610 or 1603 cm^−1^ (ELE or WBE) of alcohols or phenols of the ingredients (Scheme 1 and Scheme 2) [12], are observed at 2908, 1015 and 1562 cm^−1^ (AgNPs(ELE)), respectively, as well as at 2916, 1022 and 1610 cm^−1^ (AgNPs(WBE)), indicating the presence of the ELE or WBE in the AgNPs(ELE) or AgNPs(WBE). Thus, the ELE and WBE act as both reducing and a capping agents.

### 2.6. Ultraviolet Visible Spectroscopy (UV-vis)

**Figure 5:**
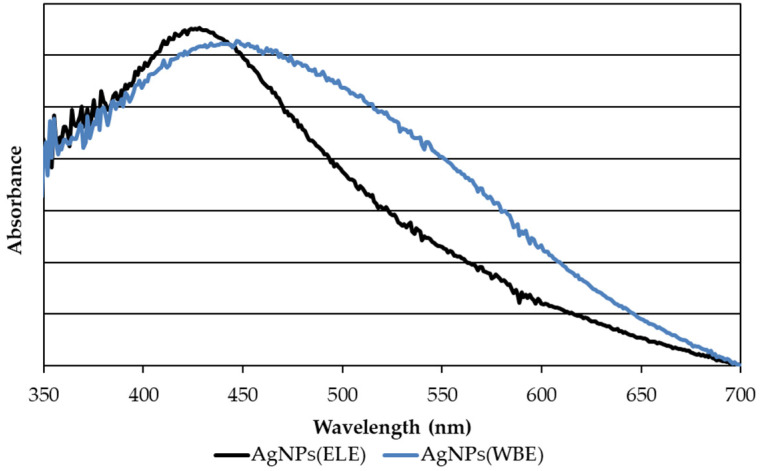
UV-vis spectra of AgNPs(ELE) and AgNPs(WBE) in double-distilled (dd) water.

UV-vis spectroscopy was implemented for the silver nanoparticles’ size determination. The UV-vis spectra in dd water solution 1 mg/mL of AgNPs(ELE) and AgNPs(WBE) showed absorbance at λmax, 428 and 447 nm, respectively (Figure 5). This band has been assigned to the collective resonance of electrons at the surface known as surface plasmon resonance (SPR) of the silver nanoparticles [23,24]. Since the size is directly related to the λmax of SPRs, particle with diameters of 40 (AgNPs(ELE)) and 60 (AgNPs(WBE)) nm were concluded [6]. These values are larger than the corresponding ones of the crystallite found from XRPD patterns, due to solvation effect.

### 2.7. Antimicrobial Study

#### 2.7.1. Determination of the Inhibition Zone (IZ) through Agar Disc-Diffusion Method

The sensitivity of the bacteria *P. aeruginosa, S. epidermidis* and *S. aureus* against the agents (ELE, WBE, AgNPs(ELE) and AgNPs(WBE)) was evaluated with an agar diffusion assay. Paper discs 10 mm in diameter were soaked in dd water solution of the agent (2 mg/mL). The discs were placed on agar plates and they were incubated at 37 °C for 20 h. The inhibition zone (IZ) was classed as the area without bacterial lawn around the disc [5,6,9,25] (Table 1). The size of the IZ around the filter paper disc indicated the sensitivity of the bacterial strain in the tested formulation [25]. Among the agents, AgNPs(ELE) gave the greatest IZ against microbial strains (Table 1). The IZs of AgNPs(ELE) against *P. aeruginosa, S. epidermidis* and *S. aureus* were 15.2, 17.0 and 15.2 mm, respectively, whereas, ELE and WBE showed no IZ (Table 1). The microbe strains were classified in three categories according to the size of the IZ caused by an antimicrobial agent: (i) strains, where the agent causes an IZ ≥ 17 mm, were classed as susceptible; (ii) those where the agent created an IZ between 13 to 16 mm (13 ≤ IZ ≤ 16 mm) were intermediate; while in (iii) those where the agent caused an IZ ≤ 12 mm the microbes were considered as resistant strains [26]. Therefore, the strains studied here (*P. aeruginosa, S. epidermidis* and *S. aureus*) responded intermediately to AgNPs(ELE), while they were considered as resistant strains towards the rest of the agents tested in this work (ELE, WBE and AgNPs(WBE)) [26].

Moreover, both ELE and WBE, alone and in combination with pHEMA, were not active at all in the disinfection process, even though their AgNPs and composite materials exhibit antimicrobial activity (Table 1). This was because plant extracts act both as reducing and capping agents in the synthesis of nanoparticles, leading to species of different sizes. The size, surface coatings or capping agents and surface charge have been identified as important factors for AgNPs efficacy. The smaller the nanoparticles, the higher their activity. Thus, when the size is < 10 nm, the activity is at a maximum. Moreover, AgNPs in the range between 10 and 15 nm, where they have higher stability, biocompatibility and antimicrobial activity, exhibit compromised activity. AgNPs over 50 nm, were not active, probably because their large dimension reduces the interaction with the bacterial surface and also impedes their internalization [27]. The variation in the sizes of AgNPs(ELE) and AgNPs(WBE) in solution (40 and 60 nm, respectively) rationalize the difference in antimicrobial activity between AgNPs(ELE) and AgNPs(WBE).

**Figure 6:**
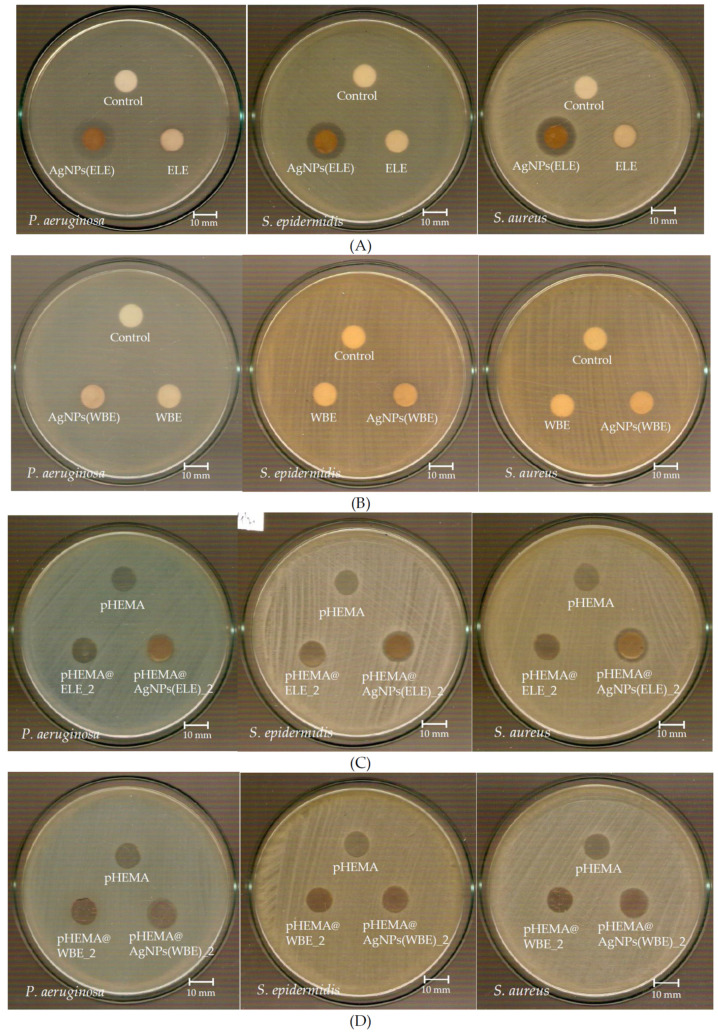
Inhibition zones of ELE and AgNPs(ELE) (**A**), AgNPs(WBE) and WBE at 2 mg/mL (**B**), pHEMA, pHEMA@ELE_2, pHEMA@AgNPs(ELE)_2 (**C**) and pHEMA, pHEMA@WBE_2 and pHEMA@AgNPs(WBE)_2 (**D**) against *P. aeruginosa*, *S. epidermidis*, *S. aureus*.

In order to check the sensitivity of bacteria to pHEMA, pHEMA@ELE_2, pHEMA@WBE_2, pHEMA@AgNPs(ELE)_2 and pHEMA@AgNPs(WBE)_2, discs with a diameter of 10 mm were placed in Petri agar plates and inhibition zones were determined after 20 h (Figure 6). The inhibition zones developed by pHEMA@AgNPs(ELE)_2 against *P. aeruginosa*, *S. epidermidis* and *S. aureus* (Table 1, Figure 6) were 12.3 ± 1.7, 13.2 ± 1.2 and 13.2 ± 1.4 mm, respectively; while no or negligible inhibition zones were developed when pHEMA, pHEMA@ELE_2, pHEMA@WBE_2 or pHEMA@AgNPs(WBE)_2 were used.

**Figure 7:**
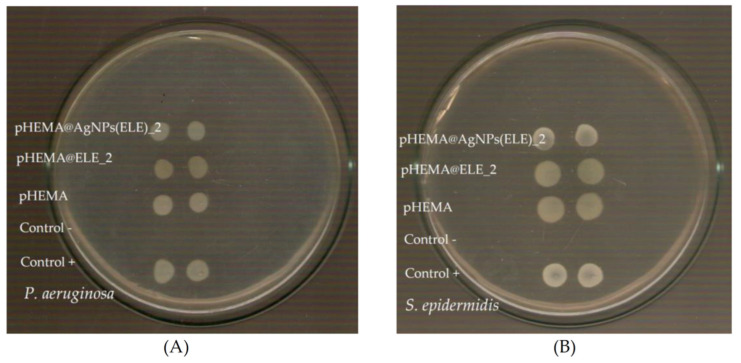
Bacterial colonies which were grown when 4 μL of the supernatant solutions of *P. aeruginosa* (**A**) and *S. epidermidis* (**B**) bacteria, which were, initially, treated with pHEMA@AgNPs(ELE)_2 discs, were inoculated in an agar plate.

For the evaluation of microbial viability, *P. aeruginosa, S. epidermidis and S. aureus* (5 × 10^5^ cfu/mL) were cultured in test tubes with broth culture medium, while pHEMA@ELE_2, pHEMA@WBE_2, pHEMA@AgNPs(ELE)_2 and pHEMA@AgNPs(WBE)_2 discs were settled in the bottom (Figure S2). Then, the tubes were stirred continuously for 20 h. The microbes’ density in the broth solutions were then determined by measuring their optical density at 620 nm and comparing that with the corresponding control solution (the corresponding one, which contains the same number of microbes, in the same volume of broth solution and was subject to no treatment with a disc). The percentage of bacterial density of *P. aeruginosa* and *S. epidermidis* upon their incubation with pHEMA@AgNPs(ELE)_2 discs was 28.3 and 6.8%, respectively (Table 1). Negligible activity in bacterial density was observed upon their treatment by discs of pure pHEMA or pHEMA@ELE_2, pHEMA@WBE_2 or pHEMA@AgNPs(WBE)_2 (Table 1). However, the optical density (OD) of the broth culture medium at 620 nm shows the microbial density; therefore, their viability still remains to be examined. In order to confirm whether the remaining microbes in broth solution were viable or not, 4 μL of the supernatant solutions of *P. aeruginosa* and *S. epidermidis* bacteria, which were, initially, treated with pHEMA@AgNPs(ELE)_2 discs, were inoculated in an agar plate. The occurrence of microbial colonies suggests the viability of the microbes after treatment and therefore a percentage reduction in the density also corresponds to the microbial viability (Figure 7). Moreover, the biomaterials reduce microbial proliferation as long as they are present in the medium, without, however, their total elimination (Figure 7).

The hydrogel of pHEMA@AgNPs(ELE)_2, which contains the AgNPs from eucalyptus leaves, exhibits superior activity against the *P. aeruginosa and S. epidermidis* in relation to those made by the rest of materials. pHEMA@AgNPs(ELE)_2 reduces the *P. aeruginosa, S. epidermidis* and *S. aureus* microbes’ proliferation.

#### 2.7.2. In Vitro Toxicity against Normal Human Corneal Epithelial Cells (HCEC)

The hydrogels pHEMA@AgNPs(ELE)_2 and pHEMA@AgNPs(WBE)_2 were tested for in vitro toxicity against HCEC cells upon their incubation for a period of 24 h. The incubation of HCEC cells in the presence of pHEMA@AgNPs(ELE)_2 and pHEMA@AgNPs(WBE)_2 discs decreased their viability by 64.4 ± 5.7 and 72.8 ± 8.5%, respectively, compared with the cells that were incubated with pure pHEMA. According to ISO 10993-5, if the percent of cell viability is higher than ≥70%, then the material should be considered as non-cytotoxic [28,29]; therefore, the materials tested in this work are considered as low or nontoxic.

## 3. Experimental Methods

### 3.1. Materials and Instruments

Dimethylsulfoxide (DMSO, Riedel-de Haen, Seelze, Germany) was used without any further purification. Silver nitrate (AgNO_3_,) was purchased from Degussa (Berlin, Germany). For lens preparation, 2-hydroxyethyl-methacrylate (pHEMA), ethylene-glycole-dimethacrylate (EGDMA, MERCK, Germany), diphenyl-(2,4,6-trimethylbenzoyl)-phosphine oxide (TPO 97%, Sigma-Aldrich, Steinheim, Germany) as well as sodium chloride (NaCl, MERCK) and hydrochloric acid (HCl 37%, MERCK) were used. Tryptone tryptophan medium, beef extract powder, peptone bacteriological and soy peptone were purchased from Biolife. Agar (Louis, MO, USA, product of Spain) and yeast extracts were purchased from Fluka Analytical (Switzerland). D(+)-glucose and di-potassium hydrogen phosphate trihydrate were purchased from Merck. Melting points were measured in open tubes with a Stuart Scientific apparatus and were uncorrected. A UV-1600 PC series spectrophotometer of VWR was used to obtain electronic absorption spectra. ATR-FT-IR spectra in the region of 4000–370 cm^−1^ were obtained with a Cary 670 FTIR spectrometer (Agilent Technologies Inc., Santa Clara, CA, USA). XRF measurement was carried out with Rigaku NEX QC EDXRF analyzer, (Austin, TX, USA).

### 3.2. Preparation of ELE, WBE and AgNPs(ELE), AgNPs(WBE)

Dry eucalyptus leaves and willow bark (4 g) were refluxed using a Soxhlet apparatus with 50 mL dd water for 3 h. The extracts ELE and WBE were filtrated off and clear filtrates were obtained. In a 250 mL beaker, 10 mL ELE and WBE and 170 mg AgNO_3_ (1 mmol) were added. The beaker was placed in a 700 W microwave oven for 2 min. Consequently, the solution was placed in the sonicator (ultrasonic) for 10 min. The solution is centrifugal at 6000 rpm for 20 min and the AgNPs(ELE) and AgNPs(WBE) were collected.

Synthesis of pHEMA@ELE_2, pHEMA@AgNPs(ELE)_2, pHEMA@WBE_2 and pHEMA@AgNPs(WBE)_2: Hydrogels of ELE, WBE and AgNPs(ELE), AgNPs(WBE) were obtained as follows: 2.7 mL of 2-hydroxyethyl-methacrylate (HEMA) was mixed with 2 mL of double distilled water (ddw), which contained ELE, WBE, AgNPs(ELE) or AgNPs(WBE) (2 mg/mL) and 10 µL of EGDMA. The solution was then degassed by bubbling with nitrogen for 15 min. TPO initiator (6 mg) was added to the solution and mixed for 5 min at 800 rpm. The solution was poured into the mold and was then placed under a 15 watt UV mercury lamp (λmax = 280 nm) for photopolymerization for 40 min. Unreacted monomers were removed, by immersing the gel in water for 15 min. Discs of 10 mm diameter were cut, and they were washed by immersion in water, NaCl 0.9%, HCl 0.1 M then again in water. The discs were then dried at 40 °C until no weight change would occur. To emphasize the composite materials, the indicator “_2” highlighted the use of 2 mg/mL of ingredients during the polymerization process of pHEMA

X-ray Fluorescence Spectroscopy: The experiments were carried out using a Rigaku NEX QC EDXRF analyzer The XRF measurements were duplicated previously reported [6] using an Am-241 radioisotopic source (exciting radiation 59.5 keV). A Si (Li) detector was used for the detection of X-ray fluorescence. In each measurement, 2000 counts were collected on the weaker Kα peak. The experiments were duplicated using a Rigaku NEX QC EDXRF analyzer.

X-ray Powder Diffraction (XRPD): This study was performed as previously reported [6]. The X-ray powder diffraction study was accomplished by a diffraction-meter D8 AdvanceBruker, from the Department of Physics at the University of Ioannina. Radiation CuKa (40 kV, 40 mA, λKα) and the monochromator system of diffracted beam were used. The X-ray powder diffraction data were collected in the area of 2θ angles between 2° and 80° using a rotation step 0.02° and time of 2 s per step. All samples measured with the above diffraction-meter were in fine-grained powder form.

Thermogravimetric Differential Thermal Analysis (TG-DTA), Differential Scanning Calorimetry (DTG/DSC): This study was conducted as previously reported [6]. The DTA/TG measurements were performed in DTG/TG NETZSCH STA 449C. The samples were placed in a platinum capsule while an-alumina was used as a reference. The rate of temperature increasing was 10 °C/minute from ambient conditions up to 500 °C under air.

### 3.3. Biological Tests

Bacterial Strains: The following bacterial strains were used in the experiment: *P. aeruginosa* (*PAO1*), *S. epidermidis* (ATCC^®^ 14990™) and *S. aureus* (ATCC^®^ 25923™) [6]. The bacterial strain *P. aeruginosa PAO1* was kindly donated by the Laboratory of Biochemistry at the University of Ioannina, Greece [6]. The biological experiments were performed in triplicate. The statistical analysis software package included in MS Office excel (Version 2107) was used for the data processing.

Effects on the growth of microbial strains: This experiment was conducted as previously reported [5,6,9,19]. In brief, bacterial strains were plated onto trypticase soy agar and were incubated for 18–24 h, at 37 °C. Three to five isolated colonies with the same morphological appearance were selected from a fresh agar plate using a sterile loop and transferred into a tube containing 2 mL of sterile saline solution. The optical density at 620 nm was adjusted to 0.1 which corresponded to 10^8^ cfu/mL [5,6,9,19].

For the determination of the inhibition zone (IZ): The procedure was performed as previously reported [5,6,9,19]. A standardized inoculum (10^8^ cfu/mL) of the microorganisms was incubated in agar plates. A cotton swab was dipped in the inoculum (10^8^ cfu/mL) of the microorganisms. The excess fluid was removed by turning the swab against the inside of the tube. The inoculum was spread evenly over the entire surface of the agar Petri dishes in three directions. Discs of pHEMA pHEMA@ELE_2, pHEMA@AgNPs(ELE)_2, pHEMA@WBE_2 and pHEMA@AgNPs(WBE)_2 with a 10 mm diameter, or ELE, WBE and AgNPs(ELE), AgNPs(WBE) at the concentration of 2 mg/mL, were placed on the agar surface and the petri plates were incubated for 20 h. In order to evaluate the viability of microbes on pHEMA pHEMA@ELE_2, pHEMA@AgNPs(ELE)_2, pHEMA@WBE_2 and pHEMA@AgNPs(WBE)_2, discs of the materials were placed in the tests tubes that contained 5 × 10^5^ cfu/mL of *P. aeruginosa*, *E. coli*, *S. epidermidis* and *S. aureus* microbes [5,6,9,19]. The optical densities of the supernatant solutions were then measured to give the percentage viability of microbes after incubation for 18–24 h [5,6,9,19]. The reference in optical measurement for percentage viability of microbes was the negative control (control(−)), i.e., the bacterial culture medium without microbes. The positive control (control(+)) was the corresponding one that contained the same number of microbes, in the same volume of broth solution, and was subject to no treatment with a disc. The percentage reduction in microbe density was calculated by the following Equation (2) [30]:

(2) $$\mathrm{Bacterial}\;\mathrm{density}\;\mathrm{reduction}\;(\%)\;=\;\frac{\;\mathrm{Optical}\;\mathrm{density}\;\left(\mathrm{OD}\right)\;\mathrm{of}\;\mathrm{the}\;\mathrm{broth}\;\mathrm{solution}\;\mathrm{at}\;\mathrm{620}\;\mathrm{nm}}{\mathrm{Optical}\;\mathrm{density}\;\left(\mathrm{OD}\right)\;\mathrm{of}\;\mathrm{the}\;\mathrm{control}\;\left(+\right)broth solution at 620 nm}\times 100$$

### 3.4. Sulforhodamine B Assay

The HCEC cells were seeded in a 24-well plate at a density of 7.5×10^4^ cells/well. After 24 h of cell incubation, pHEMA pHEMA@AgNPs(ELE)_2 and pHEMA@AgNPs(WBE)_2 were added in each well. After 24 h of incubation of HCEC cells with the discs, the discs were removed. The culture medium was aspirated and the cells were fixed with 300 μL of 10% cold trichloroacetic acid (TCA). The plate was left for 30 min at 4 °C, then washed five times with deionized water and left to dry at room temperature for 24 h. Subsequently, in each well, 300 μL of 0.4% (*w*/*v*) sulforhodamine B (Sigma) in 1% acetic acid solution was added, and then they were left at room temperature for 20 min. The dye SRB was removed, and the plate was washed five times with 1 % acetic acid before air drying. Bound SRB was solubilized with 1 mL of 10 mM un-buffered Tris-base solution. Absorbance was read at 540 nm [5,6,9,19].

## 4. Conclusions

Antimicrobial composite materials pHEMA@AgNPs(ELE)_2 or pHEMA@AgNPs(WBE)_2 for contact lenses, which are able to reduce microbial infection risks, were prepared by dispersing silver nanoparticles from eucalyptus leaves extracts into pHEMA (which is used in soft contact lenses) during the polymerization process. The most potent antimicrobial hydrogel pHEMA@AgNPs(ELE)_2 eliminates *P. aeruginosa*, *S. epidermidis* and *S. aureus* bacteria by 71.2, 93.2 and 14.7%, respectively. The oregano leaves’ extract (ORLE) was also used in the formation of silver nanoparticles AgNPs(ORLE). This was dispersed in pHEMA towards the composite material pHEMA@AgNPs(ORLE)_2. Upon the treatment of *P. aeruginosa, S. epidermidis* and *S. aureus* with pHEMA@AgNPs(ORLE)_2 discs, the percentages of bacterial elimination are 33.5, 22.3 and 40.4%, respectively; however, when pHEMA@AgNPs(WBE)_2 is used, the corresponding bacteria eliminations are 26.4, 18.4 and 17.5%, respectively [6]. Therefore, the newly developed pHEMA@AgNPs(ELE)_2 exhibits superior efficiency against *P. aeruginosa* and *S. epidermidis* than pHEMA@AgNPs(ORLE)_2 or pHEMA@AgNPs(WBE)_2. Moreover, the cell viability of human corneal epithelial HCEC cells upon their incubation with pHEMA@AgNPs(ELE)_2 discs was 64.4%, whereas the corresponding one of pHEMA@AgNPs(ORLE)_2 was 54.9%, suggesting a lower toxic effect of pHEMA@AgNPs(ELE)_2. In conclusion, pHEMA@AgNPs(ELE)_2 eliminates the *P. aeruginosa* and *S. epidermidis*, which colonize contact lenses and cause microbial keratitis, Furthermore, it exhibits low toxic effect on HCEC cells’ viability. Thus, pHEMA@AgNPs(ELE)_2 might be an efficient candidate for the development of new contact lenses that are able to inhibit colonization by *P. aeruginosa and S. epidermidis* and, in turn, reduce the risk of microbial infection.

## Figures and Tables

**Table 1:** Inhibition zones (IZs) of ELE, WBE, AgNPs(ELE) and AgNPs(WBE) at 2 mg/mL pHEMA@ELE_2, pHEMA@WBE_2, pHEMA@AgNPs(ELE)_2 and pHEMA@AgNPs(WBE)_2) against *P. aeruginosa*, *S. epidermidis* and *S. aureus*.

	*P. aeruginosa*	*S. epidermidis*	*S. aureus*
	Inhibition Zones (mm)		
AgNPs(ELE)	15.2 ± 1.2	17.0 ± 1.1	15.2 ± 1.6
ELE	ND	ND	ND
AgNPs(WBE)	11.9 ± 0.9	11.3 ± 0.6	11.0 ± 0.0
WBE	ND	ND	ND
pHEMA@AgNPs(ELE)_2	12.3 ± 1.7	13.2 ± 1.2	13.2 ± 1.4
pHEMA@ELE_2	ND	ND	ND
pHEMA@AgNPs(WBE)_2	11.2 ± 0.3	11.0 ± 1.0	11.5 ± 0.6
pHEMA@WBE_2	ND	ND	ND
	**Bacteria Viability (%)**		
pHEMA@AgNPs(ELE)_2	28.3	6.8	85.3
pHEMA@ELE_2	94.6	93.5	98.7
pHEMA@AgNPs(WBE)_2)	73.6	81.6	82.5
pHEMA@WBE_2	90.5	88.1	97.3

ND = no inhibitory zone (IZ) was developed.

## Data Availability

The data presented in this study are available in Supplementary Material.

## Supplementary Materials

The following are available online, Figure S1: TGA diagrams of AgNPs(ELE) (**A**), AgNPs(WBE) (**B**), pHEMA@AgNPs(ELE)_2 (**C**) and pHEMA@AgNPs(WBE)_2 (**D**); Figure S2: Bacteria viability of pHEMA, pHEMA@ELE_2, pHEMA@AgNPs(ELE)_2, pHEMA@WBE_2 and pHEMA@AgNPs(WBE)_2 against *P. aeruginosa* (**A**), *S. epidermidis* (**B**), *S. aureus* (C)

## Sample Availability

Samples of the compounds AgNPs(ELE), AgNPs(WBE), pHEMA@AgNPs(ELE)_2 and pHEMA@AgNPs(WBE)_2 are available from the authors.
