# Supplementary material for: Silver Nanoparticles Using Eucalyptus or Willow Extracts (AgNPs) as Contact Lens Hydrogel Components to Reduce the Risk of Microbial Infection
Source: Molecules. 2021 Aug 19;26(16):5022. doi: 10.3390/molecules26165022 (PMC8400931; doi:10.3390/molecules26165022)
Supplement: Supplementary file 1 [file molecules-26-05022-s001.zip › molecules-1334774-supplementary.pdf]

molecules

Article

Supplimentary Matterials

Silver Nanoparticles Using Eucalyptus or Willow Extracts (AgNPs) as Contact Lens Hydrogel Components to Reduce the Risk of Microbial Infection

Andreas K. Rossos [1], Christina N. Banti [1,*], Panagiotis K. Raptis [1], Christina Papachristodoulou [2], Ioannis Sainis [3], Panagiotis Zoumpoulakis [4], Thomas Mavromoustakos [5] and Sotiris K. Hadjikakou [1,6,*]

[1] Section of Inorganic and analytical Chemistry, Department of Chemistry, University of Ioannina, 45110 Ioannina, Greece; arossos@uoi.gr (A.K.R.); panagiwtisraptis93@yahoo.gr (P.K.R.)
[2] Department of Physics, University of Ioannina, 45110 Ioannina, Greece; xpapaxri@uoi.gr
[3] Cancer Biobank Center, University of Ioannina, 45110 Ioannina, Greece; isainis@uoi.gr
[4] Laboratory of Chemistry, Analysis and Design of Food Processes, Department of Food Science and Technology, University of West Attica, Egaleo,12243 Attica, Greece; pzoump@eie.gr
[5] Organic Chemistry Laboratory, Department of Chemistry, University of Athens Greece, 15571 Athens, Greece; tmavrom@chem.uoa.gr
[6] Institute of Materials Science and Computing, University Research Center of Ioannina (URCI), 45110 Ioannina, Greece
* Correspondence: cbanti@uoi.gr (C.N.B.), shadjika@uoi.gr (S.K.H.); Tel.: +302651008362 (C.N.B.), +302651008374 (S.K.H.)

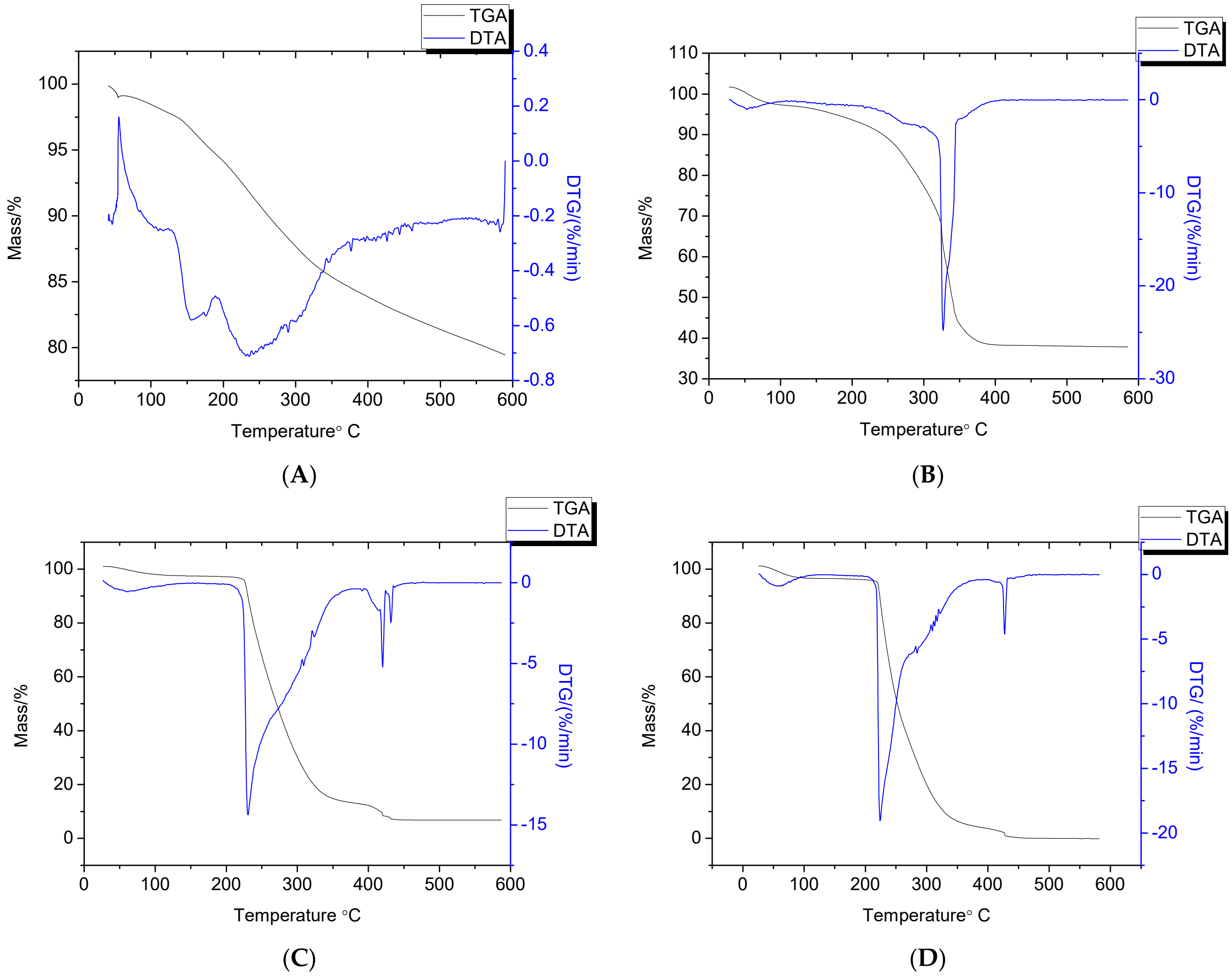

Figure S1. TGA diagrams of AgNPs(ELE) (**A**), AgNPs(WBE) (**B**), pHEMA@AgNPs(ELE)_2 (**C**) and pHEMA@AgNPs(WBE)_2 (**D**).

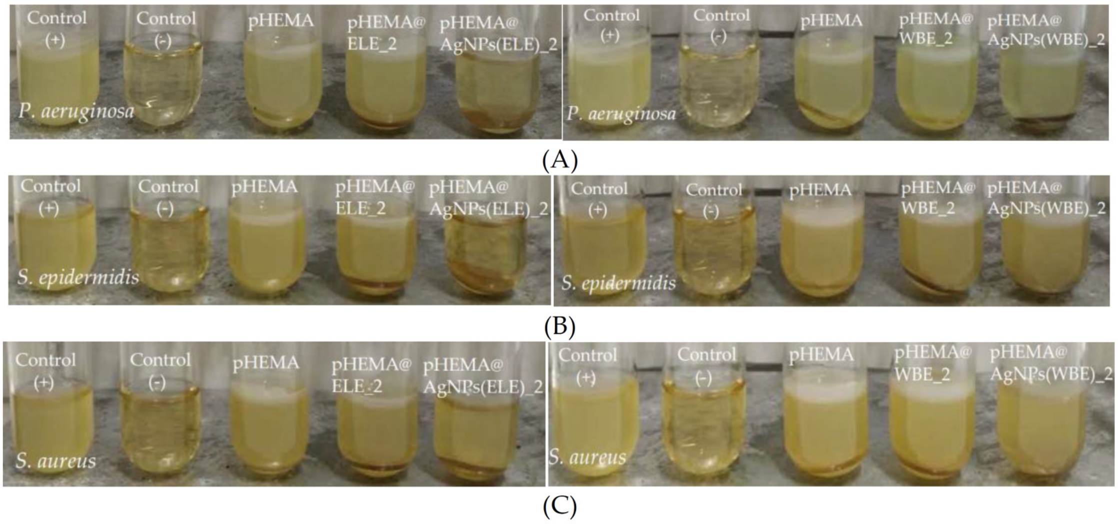

Figure S2. Bacteria viability of pHEMA, pHEMA@ELE_2, pHEMA@AgNPs(ELE)_2, pHEMA@WBE_2 and pHEMA@AgNPs(WBE)_2 against *P. aeruginosa* (**A**), *S. epidermidis* (**B**), *S. aureus* (**C**).
